# Physiotherapists’ Approaches to Patients’ Concerns in Back Pain Consultations Following a Psychologically Informed Training Program

**DOI:** 10.1177/10497323211037651

**Published:** 2021-10-07

**Authors:** Ian Cowell, Alison McGregor, Peter O’Sullivan, Kieran O’Sullivan, Ross Poyton, Veronika Schoeb, Ged Murtagh

**Affiliations:** 1 Imperial College, London, United Kingdom; 2 Brook Physiotherapy Ltd., Essex, United Kingdom; 3 Curtin University, Perth, Western Australia, Australia; 4 Bodylogic Physiotherapy, Perth, Western Australia, Australia; 5 University of Limerick, Limerick, Ireland; 6 Aspetar Orthopaedic and Sports Medicine Hospital, Doha, Qatar; 7 University of Applied Sciences and Arts Western Switzerland, Lausanne, Switzerland

**Keywords:** psychologically informed practice, communication, physiotherapy, training, qualitative, conversation analysis, United Kingdom

## Abstract

Guidelines advocate a combined physical and psychological approach to managing non-specific chronic low back pain (NSCLBP), referred to as psychologically informed practice (PIP). PIP is underpinned by patient-centered principles and skilled communication. Evidence suggests that a physiotherapist-focused style of communication prevails in physiotherapy. There is a recognized need for observational research to identify specific communication practices in physiotherapy interactions. This observational study explored the interactional negotiation of *agenda setting* following a PIP training intervention, by identifying and describing how physiotherapists solicit and respond to the agenda of concerns that patients with NSCLBP bring to primary care initial encounters. The research setting was primary care. Nineteen initial physiotherapy consultations were video-recorded, transcribed, and analyzed using conversation analysis, a qualitative observational method. These data revealed a patient-focused style of communication where trained physiotherapists demonstrated a collaborative and responsive style of verbal and nonverbal communication to solicit, explore, and validate patients’ concerns.

Guidelines advocate combined physical and psychological approaches to managing non-specific chronic low back pain (NSCLBP) (Foster et al., 2018; National Institute for Health and Care Excellence [NICE], 2016), referred to as psychologically informed practice (PIP) (Keefe et al., 2018). Patient-centered principles and communication between the patient and health care provider are central to PIP (Beneciuk et al., 2019; Keefe et al., 2018; Main et al., 2012; O’Sullivan et al., 2018). This is characterized by greater patient-physiotherapist collaboration and a facilitating style of interaction to incorporate the patient’s perspective, including their concerns, ideas, and feelings (Mead & Bower, 2000; Stewart et al., 2003). Although effective verbal and nonverbal communication skills are considered important prerequisites to such positive interaction, and to build therapeutic alliance (Pinto et al., 2012), the empirical foundations for communication practice in physiotherapy are lacking (Hiller et al., 2015).

Patient–practitioner interaction has also been characterized by more practitioner dominance (Schoenthaler et al., 2018), and existing studies of communication in musculoskeletal physiotherapy demonstrate a physiotherapist-centered style of communication where physiotherapists demonstrate difficulties balancing their own “professional” agendas with those of the patient (Cowell, McGregor, et al., 2019; Hiller et al., 2015; Opsommer & Schoeb, 2014). This physiotherapist-centered communication style has been attributed to the traditional biomedical focus of undergraduate training programs (Cowell et al., 2018; Hiller et al., 2015) and lack of postgraduate training in communication skills (Cowell et al., 2018; Daluiso-King & Hebron, 2020; Synnott et al., 2015). This is consistent with recent qualitative studies in which physiotherapists express a need for communication training (Cowell et al., 2018; Holopainen, Simpson, et al., 2020; Sanders et al., 2013; Synnott et al., 2015).

Soliciting patients’ agenda of concerns comprehensively is key to patient-centered communication (Epstein et al., 2008) and can improve patient satisfaction (Rodriguez et al., 2008). Effectively incorporating the patient’s agenda as part of a PIP interview can establish the patient’s psychosocial concerns (Keefe et al., 2018; Nicholas & George, 2011), yet it is not clear how well physiotherapists can do this in current practice.

Our previous observational study analyzed physiotherapists prior to undergoing training in PIP and demonstrated how physiotherapists do not always engage verbally and nonverbally with cues reflecting patient’s concerns about pain and incapacity (Cowell, McGregor, et al., 2019). It also highlighted that physiotherapists typically dominate the interactional agenda resulting in lost opportunities for patients to disclose and develop their agenda of concerns. To date, there has been no study exploring how physiotherapists explore patients’ concerns following PIP training.

This study explores the interactional negotiation of *agenda setting* by analyzing the extent to which physiotherapists solicit and respond to the agenda of concerns that patients with NSCLBP bring to primary care initial encounters following a training program in Cognitive Functional Therapy (CFT) (O’Sullivan et al., 2018). CFT is a psychologically informed physiotherapy-led intervention that targets physical, lifestyle, and psychological barriers to help patients self-manage low back pain (LBP) (O’Sullivan et al., 2018). Central to CFT is building a strong therapeutic alliance, which is underpinned by a reflective, empathetic, and validating communication approach (O’Keeffe et al., 2016).

The aims of this study were to identify (a) physiotherapists’ questioning strategies for soliciting patient concerns and (b) to explore how physiotherapists respond to patients’ presentation of concerns. These analyses did not focus on soliciting patients’ complete agendas of concerns but gave focus to sequences in which patients express their concerns specifically about symptom attribution and the future consequences, and their emotional agendas (Barry et al., 2000) due to loss of capacity and pain.

Conversation analysis (CA) is an inductive observational method (Maynard & Heritage, 2005) and was chosen as the research method for the study to allow for close microanalysis of the real-time interaction. The dyadic interpersonal communication model describes the dynamic interactive process that takes place between two people and has been used to characterize the interdependence of patient and provider communication (Bylund et al., 2012). CA views patient-provider communication as a dyadic process that gives equal consideration to both partners (Maynard & Heritage, 2005) and has been used previously to describe different types of interdependent patient–provider communication, which can either inhibit or promote patient participation (Collins et al., 2005; Cowell, McGregor, et al., 2019). In this study, CA was employed to explore how “concerns talk” was accomplished and co-constructed by the physiotherapist and patient. By illustrating the possible communication practices employed by physiotherapists in real-life interactions following training, this study provides empirical insights into how embedded physiotherapist-centered communication is in current practice (Cowell, McGregor, et al., 2019; Hiller & Delany, 2018; Hiller et al., 2015; Josephson et al., 2015).

## Method

### Setting

The setting for this study was two outpatient physiotherapy departments in primary care in North East London Foundation Trust (NELFT). One of the authors was employed by NELFT, and although his perspectives provided insight into the processes in the research setting and its place within the wider organization, it is acknowledged that may have had an influence on the sequences selected for inclusion in the article and the interpretation of the data. Three of the authors have a clinical and research interest in PIP and the management of LBP, and these multiple perspectives may also have consciously or subconsciously had an influence on the findings and interpretation of the data. Nineteen initial physiotherapy consultations were video-recorded (10 physiotherapists and 19 patients). The recorded assessments ranged in duration from 48 minutes to 1 hour. The lead researcher (I.C.) was present at the recorded assessments to adjust the camera as required, but he had no discussion with the physiotherapist or patient during or directly after each recorded assessment. The encounters were recorded in private treatment rooms consistent with usual practice in the research setting. The images included in this article reflect the typical physiotherapist–patient orientation during the interview phase of an initial encounter and were not manipulated for the purposes of the research.

### Participants

Ten physiotherapists (three females and seven males) who had completed a formal training program in CFT were included in this study. They ranged in years qualified from 4 to >14. The years working in a musculoskeletal setting ranged from 3 to >14. Previous postgraduate bio-psychosocial training ranged from 0 to 12 days. Twenty-three patients, reporting NSCLBP >3 months, including a range of risk profiles for developing persistent LBP, were identified in two NELFT physiotherapy departments from either the triage clinic or the musculoskeletal clinical assessment and treatment service. Two patients declined to participate due to work commitments, and two patients elected for “usual care,” as they did not want to be video-recorded, leaving 19 patients included in the study (12 women and seven men). The ages of the patients ranged from 19 to 68 years (mean of 40.8 years). The patients completed several questionnaires, which included the Ørebro Musculoskeletal Pain Screening Questionnaire (ØMPSQ; Boersma & Linton, 2005), the Roland and Morris Disability Questionnaire (RMDQ; Roland & Morris, 1983), and the STarTBack Screening Tool (Hill et al., 2008), and a measure of pain intensity on a 0–10 pain numerical rating scale (NRS). The ØMPSQ scores ranged from 83 to 150 (mean score of 112.3). The RMDQ scores ranged from 2 to 21 (mean score of 9.1). The patients’ estimated risk profile for developing persistent LBP measured on the STarTBack screening tool included eight patients at low risk, six medium, and five high. The NRS scores ranged from 4 to 9 (mean score of 6.3).

### Multifaceted Training Program

This observational study was nested in a larger study, conducted over a 3-year duration, examining the training requirements for the implementation of CFT. The multifaceted CFT training program is presented in detail in Supplemental Appendix 1. The training program included a mix of theoretical and experiential learning approaches and was informed by the physiotherapists’ own data from a pre-training observational phase (Cowell, McGregor, et al., 2019). The communication component of the training program gave explicit focus to encouraging physiotherapists to produce active displays of recipiency to facilitate patients to pursue their agenda and voice their concerns. We acknowledge that presenting post-training data from a non-randomized design such as this cannot be used to determine the effectiveness of such a training program or to interrogate the mechanisms through which clinical behavior might change. Instead, the training program simply allowed us to observe whether agenda setting and patient concerns were explored to a greater extent than that previously described (Hiller & Delany, 2018; Hiller et al., 2015; Josephson et al., 2015) and observed in our own previous (pre-training) data (Cowell, McGregor, et al., 2019).

### Analysis

All data were analyzed using CA, a qualitative data-driven inductive method based on empirical observation of communication practices (Collins et al., 2005). This method of analysis is predicated on the supposition that talk, in its ordinary and institutional form, is sequentially organized, and the meaning of each turn of talk depends on how it is understood in the next turn by the participant rather than rely on the views or interpretations of the analysts (Maynard & Heritage, 2005). There are several “intersecting machineries” of practice required for conducting the analysis (Hoey & Kendrick, 2017, p. 153), and the focus in this study was on the following aspects: *turn-taking* (how turns of talk are taken by the speakers and how this is locally managed within the talk), *structural organization* (overall “map” of the interaction in terms of analysis of different sections of interaction (e.g., opening—greeting, problem initiation, closing), *sequence organization turn-organization* (how successive turns link up to form coherent courses of action), *turn-design* (how turns are designed to perform actions, e.g., soliciting patients’ agendas of concerns), *lexical choice* (analysis of vocabulary), and *forms of asymmetries during the interaction* (Heritage, 2004; ten Have, 2004, 2007) (analysis of participation).

Initially, these post-training data were transcribed verbatim, and two members of the research team (I.C. and R.P.) independently analyzed each transcript with the accompanying video recordings. Sequences related to patients’ concerns were identified, viewed, and presented to the research collaborators to refine the direction for further analysis (Cowell, McGregor, et al., 2019). Shorter extracts of these events were then transcribed in more detail using the standardized transcription conventions for verbal and NV activity (Jefferson, 2004).^1^ NV aspects of communication during talk in which patients revealed their concerns were described in brackets. These shorter extracts were presented to the research collaborators for discussion at data workshops to support the analysis. The video recordings allowed for repeated scrutiny and provided access to the fine details of both talk and NV activity. Table 1 provides an overview of the extracts presented in this study.

**Table 1.** Summary of the Extracts Presented.

Actions	Interactional features
3.2. Soliciting and developing patients’ agendas of concerns	*3.2.1. Concern-seeking questions*
	*3.2.2. Engaging with patients’ responses following concern-seeking questions*
	*3.2.3. Formulating an interpretation of patients’ concerns—“reflecting back”*
3.3. Exploring and responding to patients’ emotional concerns	*3.3.1. Eliciting “feelings-talk”*
	*3.3.2. Providing patients with “space” for sensitive disclosure*
	*3.3.3. “Empathic formulations” conveying understanding of the client’s emotional talk*
	*3.3.4. Validating the patient’s experience*

### Question Formats

These findings illustrate particular types of questions that can be classified as either Yes/No (Y/N) questions (Raymond, 2003) or *Wh*-questions (WH-Qs; Stivers, 2010), concern-seeking questions (Robinson, 2006b), and candidate questions:

Y/N questions, or polar questions, are designed to encourage a brief “yes” or “no” response. These have been referred to as “closed” questions in that they typically limit the contributions that patients make to interactions (Boyd & Heritage, 2006). We identified two kinds of Yes/No questions: Yes/No interrogatives (YNIs) and Yes/No declaratives (YNDs), which have been differentiated in the literature in terms of how they convey the questioners’ access to information (Heritage, 2010).
*Wh*-questions are questions using words such as “what,” “why,” “when,” “who,” “where,” and “how,” and are considered less constraining than yes/no questions (Wang, 2006), providing more space for patients to design their response and describe their experience in their own terms (Peräkylä & Vehviläinen, 2003).
Concern-seeking questions are categorized on their content rather than grammatical form, in that they are explicitly formatted in ways that allowed for the relevance of concerns to be solicited (Robinson, 2006b).
Candidate questions are classified on their content and provide a model type of answer and are a common method of information-seeking (Pomerantz, 1998).

### Ethics

The local research ethics committee approved the study (reference Number: 2352), and it was successfully reviewed by the East Midlands-Nottingham 2 National Research Ethics Service (NRES) committee (14/EM/1045). All patients and physiotherapists who agreed to participate provided written informed consent prior to participation in this study, which also included authorization to use their transcripts and images in scientific articles. Video-recording live interaction has the potential to threaten privacy and confidentiality, and, therefore, the videos once collected were then kept on a password protected external hard drive, which was only available to the research team. The decision was taken to blur the faces of the participants to preserve anonymity, although it is acknowledged that this will have a detrimental impact on the presentation of the data (Parry et al., 2016), in that patients’ subtle facial expressions will be reliant to some extent on the description rather than the images.

## Results

These findings enabled insight into the identification of typical patterns of behavior and communication practices that were found in the different consultations following a multifaceted PIP training program.

### Soliciting and Developing Patients’ Agendas of Concerns

#### “Concern-seeking” questions

**Extract 1**

01 Pat: I guess it’s just my own idea about what I should

02 and shouldn’t be doing what makes it worse what

03 doesn’t

04 Phy: Yeah

05 Okay

06 Pat: Y’know should I work through pa:in

07 Phy: Yeah

08 Pat: Coz I suppose because of the damage that I had a

09 (0.2) the problem I had with there ((points to

10 left side of her back))erh how I worked through I

11 just kept going to work

12 Phy: Yeah

13 Pat: When I had th- the really bad erh slipped

14 disc or [whatever they called it]

15 Phy:       [Uhm]  [okay]

16 Yeah Yeah Yeah Yeah

17 Pat: Erm that’s and carried on then and obviously did

18 more(0.2)harm than [good] so I don’t want a

19 reoccurrence of [that]

20 Phy:      [Okay]

21 Yeah

22 **Okay and I mean in terms of do you feel your back**

23** →** **is still damaged ((clenches fist))is that**

24 **→** **something you still kind of (0.4)worried about** 

25 Pat: Erm (.)yeah I guess so I guess you don’t it it’s

26 something that stays with you so you do[::]tend

27 to worry

28 Phy:          [Ye]ah

This extract starts with the patient expressing her concerns that she is having to use her own judgment to manage her back pain and is unsure as to whether she should be working through the pain or not. This concern is built over multiple turns reflecting the patient’s perception that she caused damage previously by working through the pain: **“did more(0.2)harm than[good].”** The physiotherapist’s question that follows (Lines 22–24) acknowledges this concern and enquires as to whether the patient feels that her back is still damaged and includes a concern-seeking element: **“is that something you still kind of (0.2) worried about.”** This question is linked to the patient’s reference to “damage” earlier in the sequence. The patient’s response highlights that creating further damage is a real concern for her: **“it’s something that stays with you so you do[::] tend to worry.”** This extract provides an example of how physiotherapists topicalize and explore patients’ agenda of concerns through employing concern-seeking questions and how patients orient to the opportunities these questions present by revealing and elaborating on their concerns.

#### Engaging with patients’ responses following concern-seeking questions

**Extract 2**

01 Pat: I have spoken to my GP about it and erm they did

02 some scans and it came out that I’ve got

03 arthritis around that(.)↑area((points to lower

04 right side of back)) 

05 Phy: Okay((nodding))

06 Phy:**→** **And are you concerned about your scans?((tilts**

07 **head towards the patient and maintains eye gaze))** 

08 Pat: I am((nodding))

09 Phy: Yeah ((nodding))

10 Pat: Yes

11 Phy:**→** **Why is that**

12 (0.4)

13 Phy:**→** **[Wha-] what concerns you[about that]**

14 Pat: [Erm]           [Because what they said]

15 is arthritis((open hands))cannot be(0.2)treated

16 but you can only manage it

17 Phy: Uhum

18 Pat: And if I don’t manage it well it can get (.)

19 wo::rse

20 Phy:**→** **[Do you] you think that?**

21 Pat: [Yeah]

22 I do yes that then yeah ((nodding))

23 Phy:**→** **And how do you think you can manage it ((moves**

24 **arm to her chest and then to the patient))** 

25 **So when you say manage it what what do you mean**

26 Pat: Well by erm (0.4) by going about things the right

27 way like if you staying away from picking up

28 heavy loads (0.2) an::d not straining my back too

29 much yeah 

30 Phy: Okay ((nodding))

31 Pat: But then (.)as my daily activities (0.4) cannot

32 (.)prevent me from st- stopping that as well

33 because I’ve go children to look after [I’ve] got

34 to go to work so I’m really really concerned

35 about that I’m I’m I am concerned ((nodding))

This extract starts with the patient reporting that a previous magnetic resonance imaging (MRI) scan had revealed degenerative changes. The physiotherapist’s concern-seeking question—**“And are you concerned about your scans”**—is *and*-prefaced keeping the scan as the topical focus. Although in terms of its content, this question is explicitly formatted as a concern-seeking question, it is grammatically a YNI, which generally functions by inviting agreement or disagreement, and produces just a simple **“I am”** confirmation from the patient. The physiotherapist’s less constraining *WH*-Q that follows in Line 11, **“Why is that,”** suggests that the initial concern-seeking question appeared to solicit more than the minimal response it received. The question is met with initial patient hesitation prompting the physiotherapist to reformulate the *WH*-Q more explicitly around the patient’s concerns: **“what concerns you[about that].”** This *wh*-prefaced concern-seeking question is designed to explore further the patient’s concerns, and Line 14 marks the start of a more elaborate patient response over two turns, in which the patient reveals her perception that the condition is incurable, requiring careful management to prevent future deterioration (Lines 14–19): **“Because what they said is arthritis cannot be (0.2) treated but you can only manage it.”** The patient accounts for her negative perspective using a third-party attribution: **“what they said,”** and the physiotherapist picks up on this and enquires at Line 20 as to whether this is also the patient’s perspective: **“[Do you] you think that?”**. Such *think*-formulated questions are common in these findings and appear to be used here as a resource to invite the patient to offer their own ideas on attribution or management. The patient’s response (Lines 21–22) suggests emphatically that this is her main concern. The physiotherapist’s *WH*-Q that follows (Lines 23–25) and again includes **“you think”** is contingent on the patient’s response in the previous turn and designed to better understand the patient’s views on what she means by “managing” the condition. The patient’s response (Lines 26–29) reveals that avoidance is her strategy for managing her condition. The physiotherapist’s acknowledgment token **“Okay,”** while simultaneously nodding her head, provides space for the patient to continue, which she does, revealing that the occupational demands of nursing mean that she cannot always avoid physical stress and has to work to provide for her family. This full expression of her concerns for the future and providing for her children is revealed explicitly at the end of the sequence (Lines 31–35): **“I’m really really concerned about that I’m I’m I am concerned.”** In this extract, the physiotherapist designed her turns (initial and follow-up concern-seeking questions, prompting *WH*-Q’s and *think*-formulated questions) based on the patient’s prior talk, providing a sequential relevance for the patient to elaborate on her concerns.

#### Formulating an interpretation of patients’ concerns—“reflecting back”

**Extract 3**

01 Pat: = now I feel that(0.4)if I do that it might

02 make it worse so:

*Lines omitted Pat makes a joke about not being fit*

03 Phy: So(.)have you stopped badminton because your

04 worried about it that it might make it worse or

05 that you tried it and you struggled to play

06 Pat: Erm::: (0.2) the worry of it=

07 Phy: [Right]

08 Pat: [=making it worse]

09 Phy:**→** **So it’s not like you’ve done it experienced it**

10 **making it worse it’s just that you’re worried**

11 **that if you do it might [make it worse]so**

12 Pat:     [Yeah so yeah]

13 Phy: And what do you think might happen if you did

14 if you played badminton then

15 Pat: Just like an increased level of (0.2) discomfort

16 and pain with it which (0.2) obviously with work

17 (gestures right hand to the physiotherapist))

18 Phy: ↓Yea::h ((slowly nods head))

19 Pat: Y’know((nods head))

20 Phy:**→** **So you’d be worried about it increasing the**

21 **pain and therefore limiting your(.)your**

22 **[work]((gestures with left hand))**

23 Pat: [Definitely] yeah((gestures with right hand))

24 Like I say with the work situation is (0.2) you

25 only get paid [when your there so it’s]

26 Phy:**→**     **[Takes priority for you]**

27 Pat: Of course yeah

**Figure 1.**
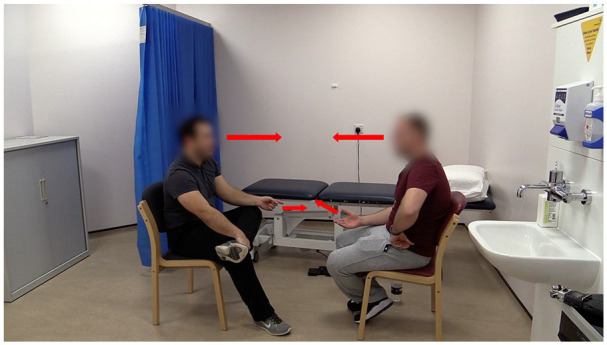
The arrows denote synchronized hand gestures and aligned eye gaze (physiotherapist on the left).

This extract starts with the patient describing the impact of his LBP and how he now avoids sports he previously enjoyed because of concerns about making the condition worse. The physiotherapist’s *so*-prefaced YNI question that follows includes candidate answers (Lines 03–05) and appears to be positioned and constructed to better understand whether his avoidance of sporting activities is based on worry/expectation of pain or the actual experience of activity-provoked pain. The patient’s response, **“the worry of ..making it worse,”** is followed by the physiotherapist’s *so*-prefaced formulation^2^ to summarize the patient’s response (Lines 09–11). Formulation-type interpretations, as illustrated here, where the physiotherapist interprets and “reflects back” what the patient has said, are common in these findings. Such formulations have a preference for agreement, which it receives in the form of an overlapping affiliating response from the patient: **“[Yeah so yeah].”** The physiotherapist’s follow-up *WH*-Q is *and*-prefaced and *think*-formulated and sustains the focus on the patient’s prior responses and targets his perception of consequences: **“And what do you think might happen if you did if you played badminton then.”** The patient responds by referring to an increase in pain and its effect on his capacity to work. The physiotherapist’s intonational shift in pitch and stretched acknowledgment token **“↓Yea::h”** marks the patient’s talk about work impact as significant information. The physiotherapist’s understanding of the patient’s work concern is made more explicit with a further *so*-prefaced formulation, **“So you’d be worried about it increasing the pain and therefore limiting your (.) your [work],”** which again receives a strong affiliative response with overlap, **“Definitely] yeah.”** The synchronized hand gestures in this turn seem to convey, and reinforce, a sense of collaborative understanding and affiliation (see Figure 1). This extract highlights how formulations can occasion extended concerns-talk and function to preserve cumulative understanding.

### Exploring and Responding to Patients’ Emotional Concerns

#### Eliciting “feelings-talk”

**Extract 4**

01 Pat: So I’m just thinking (.)I’ve got to live my whole

02 life with (.) severe back pain

03 Hhh and erm::((looks away from the

04 physiotherapist)) 

05 Phy: **→** **>So how did you feel at that point when you were**

06 **told that<**

07 Pat: ^o^I fe- I felt^o^((returns eye gaze to the

08 physiotherapist))(0.6)wow this is something I’ve

09 got to live with((moves both hands to

10 physiotherapist))(0.4) somehow I’ve go to live

11 this for the rest of my life

12 Phy: ((Nods))

13 Phy:**→ And was that (.) frustrating or was it upsetting** 

14 **[or::]**

15 Pat: [There’s]frustration definitely frustrating=

16 Phy: Yeah

17 Pat: =definitely erh:::(0.4)worrying ((moves body

18 towards the physiotherapist))

19 Phy: Worried

20 Pat: Yeah

At the start of this extract, the patient reports that his spinal consultant has suggested that he should expect a future of ongoing pain: **“life with (.)severe back pain.”** The physiotherapist’s *so*-prefaced *WH*-Q (Lines 05–06), **“So how did you feel,”** makes “feelings-talk” a relevant next action. The patient orientates to the relevance of talking about his feelings by incorporating, **“I felt”** at the start of his turn, but the hitches and intra-turn pauses suggest some difficulty producing the turn. The self-repair^3^ from **“**^o^ **I fe- I felt**^o^ **wow this is something I’ve got to live with”** to **“somehow I’ve go to live with this for the rest of my life”** appears to reflect the distress of the prognosis. The physiotherapist’s *and-*prefaced question that follows (Lines 13–14) keeps the topic on track and incorporates a candidate answer, **“And was that(.)frustrating or was it upsetting or::,”** which makes relevant further emotional disclosure. Providing almost a model of the type of answer is a way of displaying and having knowledge of the circumstance and perhaps creates an environment for disclosure by again making the patient’s feelings relevant and understandable. Although patients do not always orient to the proffered suggestions, it does provide an opening gambit for feelings talk and provides space for patients to refine, correct, or add their own dimension. In this extract, the patient affiliates to the frustration but introduces his own dimension, **“worrying.”** This extract provides illustration of how physiotherapists’ questions were designed to elicit patients’ emotional concerns and make feelings-talk a relevant topic.

#### Providing patients with “space” for sensitive disclosure

**Extract 5**

01 Pat: Erm (0.2) yeah I think its affecting me life

02 quite a lot because it’s very depressing(0.2)when

03 you can’t get away from a pain((looks down to the

04 floor and then back to physiotherapist)) 

05 Phy:**→** ^O^**Uhm**^O^**((slow nodding))((eye gaze and bodily**

06 **orientation toward the patient))** 

07 Pat: And even like (0.2) things that people take for

08 granted y’know I’ve got two grandsons (0.2)they-

09 they done all the sports boxing football do

10 everything=

11 Phy:**→** ^O^**Uhm**^O^ **((mini nodding))**

12 Pat: =you wanna go and watch them now everyone else

13 goes and stands on the field there’s me I’ve got

14 my chair me me painkillers a blanket in case I

15 get cold >like an old girl< 

16 Phy:**→** **[**^o^**Yeah**^o^**]((mini nodding))**

17 Pat: [You know]what I mean

18 Erm it’s just(.)inconvenient and then I jump up

19 coz their got a goal[[((smiles))]]now I’ve gotta

20 go home and go to bed 

21 Phy:      [^o^He he he^o^] ((nodding))

**Figure 2.**
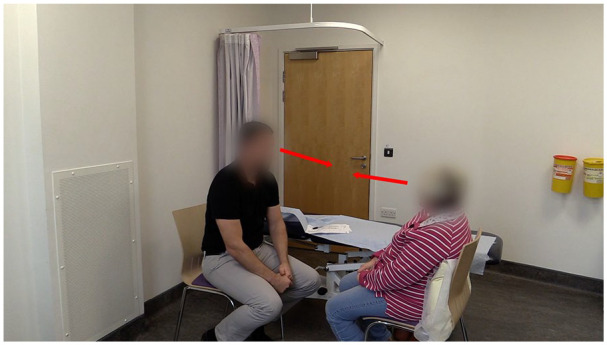
Physiotherapist’s body orientation and eye gaze toward the patient communicating engagement.

This extract starts with the patient describing the impact of her back pain and includes an explicit reference to her emotional distress: **“it’s very depressing (0.2) when you can’t get away from a pain=.”** The patient’s emotional stance is also captured by her withdrawal of eye gaze from the physiotherapist to the floor (Lines 03–04). The physiotherapist’s minimal continuer **“**^O^ **Uhm**^O^ **”** (Line 05) and slow nodding of the head comes at a potential completion point of the patient’s turn and signals that the patient has space to continue to talk about her feelings. The empathic continuers **“**^O^ **Uhm**^O^ **”** and acknowledgments **“**^O^ **Yeah**^O^ **,”** as illustrated here (see also Extract 5), are characterized by low volume and appear to resonate with the client’s emotional disclosure and allow the patient space to describe, over several turns, the functional impact of living with persistent pain. Of note is how the physiotherapist maintains his eye gaze and bodily orientation toward the patient throughout the sequence (see Figure 2), displaying that the patient is the dominant focus at this sensitive moment. Generally in these findings, and again illustrated here, the physiotherapists seemed to demonstrate awareness of the potential intrusiveness of documentation in these sensitive moments by not shifting their focus of attention to the documentation (see Figure 2). This extract provides illustration of how physiotherapists’ minimal and quiet empathic receipts allow the patient space to disclose their distress and how coordinated body orientation signals their attention and engagement with the patient.

#### “Empathic formulations” conveying understanding of the client’s emotional talk

**Extract 6**

01 Pat: Yeah so I I almost like know in the long term

02 (0.4) h::: erm ((looks to the window)):hhh that

03 erh hhh: it’s gonna get worse and I don’t- it’s

04 gonna disable me((looks sad)) 

05 Phy: ((mini nodding throughout whilst looking at the

06 patient))

07^ o^Okay^o^ so that’s why [you-]

08 Pat:     [Cos it] has done

09 it to people I have seen people (0.2)I have seen

10 >my job doesn’t< does I love MY JOB but it it

11 doesn’t help with with [with my erh] with my erm 

12 cos erh I do twelve hour shifts

13 Phy:          [Uhum]((nodding)) 

14 Yeah

15 Pat: And it’s really intensive an it’s hard

16 Phy: Yeah

17 Pat: Yeah (0.4) but erh::: it doesn’t make any favours

18 for my back

19 Phy: Mhm

20 Pat: Yeah

21 Phy: Okay

22 Pat: And I I’ve done it for, for couple of years now

23 that’s the only best thing I know how to do 

24 Phy: Yeah

25 Pat: Yeah so

26 Phy: **→** **Okay so was it it’s a bit of a scary outlook for**

27 **you=**

28 Pat: Yeah ((nodding))

29 Phy: **=at the moment ((nodding))**

30 Pat: Yeah yeah I have seen nurses who have erm who are

31 in care homes ((sad face))

32 Phy: ^o^Mhm^o^

33 Pat: Cos I’s when I was training I have seen them and

34 they are young and erh disabled and erh you talk

35 to them an it’s really sad an I see myself going

36 that way

37 Phy: ^o^Okay^o^ ((higher pitch))

**Figure 3.**
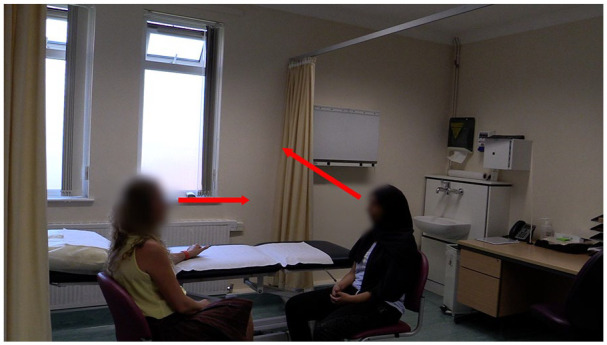
Patient looks away toward the window and the physiotherapist maintains eye contact with the patient.

This extract starts (Lines 01–04) with the patient describing her concerns for the future, predicting with some certainty that her back pain will deteriorate and ultimately **“disable”** her. This patient turn is punctuated with pauses, exaggerated in- and out-breaths, facial expressions of sadness, and withdrawal of eye gaze from the physiotherapist to the window. The physiotherapist maintains her eye gaze and bodily orientation toward the patient throughout (see Figure 3). The physiotherapist’s gaze and body orientation combined with minimal head nodding allow the patient to continue and express her concerns. The physiotherapist’s *so*-prefaced turn that follows (Line 07), **“so that’s why [you],”** is cut off by the patient’s overlapping turn in which she accounts (offers an explanation) for her concern, **“Cos it] has done it to people I have seen people . . ,”** and this sequence includes further patient elaboration, over several turns, of the physical demands of the job on her back and how nursing is the only job she is qualified to do. The physiotherapist’s empathic formulation (Lines 26–27 and 29) that follows, **“so was it it’s a bit of a scary outlook for you at the moment,”** frames the patient’s vision for the future as “scary.” This empathic formulation produces strong patient agreement, **“yeah yeah” ((nodding)),** followed by an extended emotional response, which includes an account where she has witnessed young nurses becoming disabled (Lines 33–36). This extract provides an illustration of how physiotherapists use empathic formulations to represent the patient’s emotional experience and allow patients to build and elaborate on their emotional concerns.

#### Validating the patient’s experience

**Extract 7**

01**** And with damage((uses both hands))what do you

02 feel(.) what does that mean to you what do you

03 feel 

04 Pat: I I guess I don’t wan- h:: (0.6) I wouldn’t like-

05 erh well I wouldn’t like to have another

06 operation

07 Phy: No((head nodding))

08 **→ Yeah which is fair enough ((**^o^**he he he**^o^**))**

09 Pat: I mean it worked really well [but] I don’t want

10 to put myself [or my family through] me having

11 another operation 

12 Phy:       [Yeah]

13     [No ((nods head))]

This extract is a continuation of Extract 1 and starts with the physiotherapist (Lines 01–03) attempting to elicit why the patient feels her back is damaged. The response produces the patient’s main concern, **“Well I wouldn’t like to have another operation,”** a reference to the patient’s previous surgery. The physiotherapist responds with the simple acknowledgment, **“No,”** while simultaneously nodding her head, which demonstrates affiliation with the patient’s concern. This affiliation is made more explicit by the physiotherapist’s attempt to further legitimize the patient’s concern in Line 08, **“Yeah which is fair enough.”** This validating response is followed by further patient elaboration as she builds her concern in the next turn, emphasizing the stress that another operation would place on her and her family. Further minimal agreement tokens, **“Yeah”** and **“No,”** acknowledge the patient’s concerns (Lines 12–13), which are embodied by the physiotherapist simultaneously nodding her head.

## Discussion

### Overview

In these findings, the physiotherapists “actively” solicited and explored patients’ concerns and were responsive to patients’ implicit and explicit cues. The key aspects of these findings will now be discussed in more detail.

### Exploring Patients’ Concerns

Patients’ concerns were prioritized explicitly by the frequency of concern-seeking questions (Extract 1, Lines 23–24). Physiotherapists do not always engage with patients’ responses following concern-seeking questions, preferring to pursue their own agenda (Cowell, McGregor, et al., 2019). By contrast, here the physiotherapists consistently engaged with patients’ responses by employing secondary questions. These secondary questions were typically *WH*-Q’s, for example, **“Why is that”** (Extract 2, Line 11), and appeared to be seeking extended rather than short and unelaborate responses (Fox & Thompson, 2010), allowing patients’ initial concerns to be explored. These *WH*-Q’s were often *think*-formulated probing patients’ attribution and management ideas (Extract 3, Lines 13–14). Such *think*-formulated questions are consistent with exploring patients’ illness experience (Stewart et al., 2003) and may help physiotherapists identify gaps between their own and the patient’s understanding of their back pain. This is important as any mismatch between the physiotherapist and patient, in terms of expectations and beliefs about their back pain and treatments, is recognized as a barrier to delivering effective PIP and patient-centered practice (Ozer et al., 2000).

Physiotherapists preserved the patient’s concerns-displays as a legitimate topic for discussion by providing minimal responses, such as **“Yeah,” “Uhum,”** combined with head nodding and maintaining eye gaze, demonstrating that the patient would continue to talk (Schegloff, 1982).

The physiotherapists’ responsiveness was also reinforced by their attempts to interpret patients’ previously expressed concerns. Such “reflecting back” or formulating the meaning of the patients’ earlier statements (Antaki, 2008; Heritage & Watson, 1979) is consistent with recommended patient-centered interviewing techniques (Hashim, 2017; Main et al., 2010). While formulations can close topics (Heritage & Watson, 1979), in these findings, the formulations “fixed” the topic on the patients’ concerns, and patients mostly oriented by providing elaboration (see Extract 3, Lines 23–25), as previously observed in psychotherapy, medical, and physiotherapy data (Beach & Dixson, 2001; Bonnin, 2017; Cowell, McGregor, et al., 2019).

### Exploring Patients’ Emotional Concerns

There is good evidence that emotional distress impedes recovery for patients with persistent back pain (Crombez et al., 1999; Foster et al., 2008; Pincus et al., 2002). Despite recommendations that physiotherapists identify and target these risk factors (Foster et al., 2018; NICE, 2016), there is little interactional evidence supporting physiotherapists’ willingness or ability to do so. The limited available data suggest that physiotherapists rarely question patients with LBP on the emotional impact of their condition (Roussel et al., 2016), despite patients seeing this as key to “good” clinical interaction (Laerum et al., 2006). While physiotherapists recognize the importance of addressing patients’ emotional factors, they often feel uncomfortable addressing sensitive topics, reporting a lack of training and guidance in this regard (Cowell et al., 2018; Fritz et al., 2018; Holopainen, Simpson, et al., 2020). Recent evidence has demonstrated how some physiotherapists acknowledge but fail to explore patients’ emotional concerns in preference for pursuing their own agenda (Cowell, McGregor, et al., 2019; Josephson et al., 2015).

In contrast, these findings strongly suggest that physiotherapists viewed patients’ emotional concerns as an integral aspect of physiotherapy interaction. They frequently initiated feelings talk by employing *feelings*-formulated questions (Extract 4, Lines 05–06), or candidate questions, which included emotions as model answers (Extract 4, Lines 13–14), making patients’ feelings a relevant next topic. Patients typically orientated to the opportunities to reveal their emotional concerns, displaying how these were interactively produced in a physiotherapy context. The patients’ affective displays were marked here as sensitive through hesitations, hitches and perturbations, laughter particles, prosodic shifts in pitch and volume, and through emotional expressions such as **“upset,” “sad,” “depressing.”** Multimodal communication is particularly important in emotional displays (Peräkylä & Ruusuvuori, 2012), and patients disclosed their emotions not exclusively through their talk. Facial expressions (sad face) combined with NV behaviors (withdrawing eye gaze) appeared to reinforce patients’ emotional stance. Presentation of emotional concerns may be built from weak hints to more explicit emotional expressions (Mellblom et al., 2016), and in these findings, patients typically built their emotional concerns over a number of turns facilitated by the physiotherapists’ empathic responses.

The physiotherapists’ sensitivity and engagement with the patient in these emotional sequences was reflected by their minimal responses, which had a different quality and seemed to resonate with patients’ quiet emotional disclosure (Fitzgerald & Leudar, 2010), for example, **“**^o^ **Mhm**^o^ **,” “**^o^ **Yeah**^o^ **,” “**^o^ **Okay**^o^ **.”** These unobtrusive responses, combined with minimal and slow nodding of the head, appeared to orient to the physiotherapists’ expectation for more sensitive disclosure and providing patients with the space for this.

The physiotherapists’ responsiveness to patients’ emotional talk was also demonstrated by their attempts to represent the patients’ emotional experience by employing *empathic* formulations (Fitzgerald & Leudar, 2012). These formulations provided a sensitive way to orient to the patient’s emotional talk, for example, **“a scary outlook for you”** (Extract 6, Lines 26–27). Empathic validation was also demonstrated by the physiotherapists’ explicit statements, for example, **“[that’s understandable],”** which legitimized patients’ emotional concerns.

### Nonverbal Behaviors

Very little focus has been given to NV communication in physiotherapy interaction (Parry & Brown, 2009). In our previous observational study, prior to the CFT training intervention, we observed how some physiotherapists demonstrated a lack of direct body orientation and abrupt withdrawal of eye gaze, communicating a reduced state of engagement with the patient (Cowell, McGregor, et al., 2019).

In contrast, these findings were characterized by the physiotherapists’ consistent body orientation, eye gaze, and hand gestures toward the patient, communicating a framework of engagement (Robinson, 2006a). Such behaviors communicated that the patient was the dominant focus and enabled physiotherapists to detect patients’ facial expressions and bodily displays expressing their symptoms and distress (Heath, 2002). Patients also displayed their orientation to these nonverbal displays through their own body behavior, with illustrations of synchronized patient-therapist hand gestures (see Extract 3; Figure 1) and reciprocal head nodding.

Using documentation during the interaction is a widely recognized barrier to effective communication (Cowell, McGregor, et al., 2019; Schoeb & Hiller, 2018). In these findings, the physiotherapists’ sensitivity to the intrusive nature of documentation was manifest in their body comportment and eye gaze away from the documentation and toward the patient (Robinson, 1998). This is consistent with self-promotional goals theory (Goffman, 1979; Jones & Pittman, 1982) and wanting to communicate an expression of interest to the patient.

We have observed in these findings how key communication features could influence the disclosure of patients’ expression of concerns. Such verbal and NV communication features provide tangible empirical examples of the recommended skills of patient-centered communication required to develop the therapeutic relationship (Bedi & Duff, 2014) and are at the heart of PIP approaches (Main et al., 2012; O’Sullivan et al., 2018).

## Strengths and Limitations

These findings seem observably different from data from previous studies, including our own pre-training data (Cowell, McGregor, et al., 2019; Hiller et al., 2015; Josephson et al., 2015); however, the design precludes specific conclusions being made about the effects of the training program. Consequently, further research using quantitative methods is needed to determine the effectiveness of this training model in changing physiotherapists’ communication practice. Future work might also consider including patients with higher levels of disability as it is acknowledged that patients at high risk of poor outcome have higher levels of emotional distress (Hill et al., 2008; Main et al., 2012). Validation of the findings was strengthened by a strong commitment to naturalistic description of the interaction and ensuring that the researchers’ analyses were aligned to how the interactants themselves locally interpreted the interaction, by closely analyzing how the next speaker treats the preceding action (Silverman, 2004). However, the video recordings were undertaken in two settings in primary care only; therefore, no representation of practice can be claimed. It is also acknowledged this is a relatively small sample size and, therefore, the findings discussed are suggestive of the *possible* types of practices employed by physiotherapists in real-life interactions, yet perhaps not representative of *all* physiotherapists’ practices following this type of PIP training (Peräkylä, 2011). It is recognized that using video recording might have had an influence on how participants behave (Parry, 2010) and that the presence of the lead author may have disrupted natural interaction. This PIP training program was experiential, extensive, and multi-staged over an extended period of time, which has clear practical implications in terms of the resources needed for wide-scale implementation. Health reform is also needed to better align funding with evidence-based practice (Foster & Delitto, 2011; Keefe et al., 2018), as short appointment times make delivering PIP challenging (Cowell, O’Sullivan, et al., 2019; Holopainen, Piirainen, et al., 2020). We also observed in these findings how physiotherapists integrated documentation tools to limit their intrusive nature when addressing patients’ concerns. Being able to integrate physiotherapy documentation to limit its intrusive nature when addressing patients’ concerns may be difficult in current practice given an increasing move toward the use of computerized documentation (Schoeb & Hiller, 2018). It has been suggested that the structure of documentation tools may need reconsideration so that they align more seamlessly with the flow of the conversation (Schoeb & Hiller, 2018).

## Practice Implications

We observed how key verbal and NV communication features helped solicit and validate the disclosure of patients’ concerns. These findings may help physiotherapists to reflect on the elements of communication, such as levels of bodily engagement, actively listening, accurately summarizing and empathizing with patients’ expressed concerns, not being incorporated into their current practice. This process of reflection on practice may be enhanced by expert observation and feedback on physiotherapists’ communication practice, as used in this training program. These findings illustrate the importance of multimodal communication in patients’ emotional displays and demonstrate how physiotherapists’ empathic responses are important in allowing patients to disclose and build their emotional displays.

## Conclusion

Following a training program in CFT, we observed how physiotherapists were prepared to share control of the interactional agenda and prioritize patients’ concerns. The physiotherapists were responsive to patients’ “talk,” employing key verbal and NV communication behaviors to support patient disclosure and allow the exploration and validation of patients’ concerns. This contrasts with recent studies that have consistently demonstrated a more physiotherapist-focused style of communication, including with the same physiotherapists prior to this training. This suggests that the communication behavior of physiotherapists may be amenable to change.

## Supplemental Material

sj-pdf-1-qhr-10.1177_10497323211037651 – Supplemental material for Physiotherapists’ Approaches to Patients’ Concerns in Back Pain Consultations Following a Psychologically Informed Training Program Click here for additional data file. Supplemental material, sj-pdf-1-qhr-10.1177_10497323211037651 for Physiotherapists’ Approaches to Patients’ Concerns in Back Pain Consultations Following a Psychologically Informed Training Program by Ian Cowell, Alison McGregor, Peter O’Sullivan, Kieran O’Sullivan, Ross Poyton, Veronika Schoeb and Ged Murtagh in Qualitative Health Research

## Notes

1. Transcription convention used in extracts: Like Underlining denotes accentuated intonation (1.6) Indicates a pause timed in seconds and tenths of seconds (.)  Indicates a pause of less than 0.4 of a second [Or::] Square brackets indicate the point [Yeah] where overlap begins and ends again= Equal signs indicate that there is no =so space between utterances mi- A dash following a word or part of a word indicates that the last sound has been cut short or:: Colons indicate stretched sounds °That (.) I don’t knoh:w.° Speech contained within degree symbols is notably quieter than the surrounding speech ↑  An upward facing arrow indicates a marked step up in pitch ↓  A downward facing arrow indicates a step down in pitch ((Writing in chart)) Double brackets contain descriptions of nonverbal actions > < Indicates speeding-up .hhh In-breath hhh Out-breath ?  Indicates a rising intonation W (h) ord Indicates laughing while talking

2. **Formulations** “reflect back” an interpretation of talk (Heritage and Watson, 1979).

3. **Self-repair** is a self-correction mechanism initiated by the speaker (Sidnell, 2010).
