# Supplementary material for: Physiotherapists’ Approaches to Patients’ Concerns in Back Pain Consultations Following a Psychologically Informed Training Program
Source: Qual Health Res. 2021 Oct 7;31(13):2486–501. doi: 10.1177/10497323211037651 (PMC8579327; doi:10.1177/10497323211037651)
Supplement: sj-pdf-1-qhr-10.1177_10497323211037651 – Supplemental material for Physiotherapists’ Approaches to Patients’ Concerns in Back Pain Consultations Following a Psychologically Informed Training Program [file sj-pdf-1-qhr-10.1177_10497323211037651.pdf]

Appendix 1: Description of the CFT Training programme

This table has been reproduced (with minor modification) with permission from John Wiley & Sons Ltd (Cowell et al., 2019)

Training mode	Description of content	Purpose
**Web-based resources**	www.pain-ed.com this website includes patient and clinician stories as well as up to date research articles.	To bridge the gap between science and clinical practice and provide updates in pain research and current best evidence practice. To provide the latest research on behavioral psychology, neuroscience and the development of disability.
**E-book**	Evidenced-based operational definitions for the key bio-psychosocial (BPS) domains/constructs represented in the Cognitive Functional Therapy (CFT) multi-dimensional clinical reasoning framework (MDCRF).	To compliment the formal training programme operational definitions were provided to enhance understanding of terminology and enhance knowledge of key domains.
**Educator-led teaching Educator-led teaching (3-days)**	**MDCRF**<br>The multi-dimensional factors associated with LBP, including pathoanatomical, physical, psychological, (cognitive and emotional), social, life-style, and general health were covered.	To develop awareness of the broad framework and to identify underlying causal mechanisms and stratify LBP patients from a multi-dimensional perspective.
	**Communication**<br>Conducting the interview process and how to effectively facilitate patient disclosure. Developing a strong therapeutic alliance by an open, reflective, empathetic, and validating communication style. Helping patients to make sense of their pain, develop effective pain control strategies and adopt healthy lifestyle behaviors. Collaborative goal setting and determining patient readiness to engage in the process. 'Giving' effective reassurance.	To enhance knowledge and develop awareness of best evidence guidelines for communication practice. To develop effective communication awareness and knowledge including: utilising empathy, reflective questioning, and motivational interviewing techniques in order to listen to the patient's story and explore their concerns, pain beliefs, fears, coping strategies, life stresses, psycho-social factors, pain behaviour, impairments and goals and deliver effective reassurance targeted to patient's concerns.
	**CFT intervention**<br>Developing CFT interventions that target maladaptive motor control/ movement impairments and maladaptive cognitive and lifestyle factors.	To develop competency in targeted interventions using CFT as an approach.
**Masterclass observation (4 hrs.)**	Observation of a CFT educator assessing and treating four "live" patients.	To provide opportunities for observing and modeling experienced clinicians.
**Problem based learning (PBL) (6 hrs.)**	1. Identifying BPS domains.	To encourage confidence and accuracy in identifying the broad BPS domains.
**PBL (1 hr.)**	2. Identifying pain characteristics.	To develop the knowledge and competency identifying different pain characteristics and behaviours suggestive of underlying pain mechanisms.
**PBL (1 hr.)**	3. Motivational interviewing (MI), building therapeutic relationships and promoting behavioural change.	To consolidate the theoretical lecture on MI and highlight the process and spirit of MI.
**PBL (5 hrs.)**	4. Communication data session where the physiotherapists' actual recordings (accompanied by the transcript) of consultations from the pre-training phase were played to the group, and the interactional trajectory of the conversation were discussed. The extracts gave particular focus to facilitating patient disclosure, including their full expression of concerns and perceptions about symptom attribution. It also focused on 'giving' effective reassurance. All extracts highlighted verbal and non-verbal features of communication practice.	To enhance awareness of effective communication practice and promote reflection on the physiotherapists' actual communication behaviours.

**PBL (2 hrs.)**	5. Providing individualised pain explanations *'explain pain'*	To develop competency in changing maladaptive illness beliefs and reconceptualise patients' understanding of pain, and better equip patients with a personalised understanding of their pain disorder.
**PBL (2 hrs.)**	6. Developing CFT interventions	To develop skills in hands-on feedback and movement re-education skills to effectively teach functional behavior change strategies.
**Experiential learning (6-Mths.)**	Individual clinical observation and educator feedback in the physiotherapists' own clinical environment, as well as 1:1 video review sessions of the baseline recorded assessments.	To enhance experiential learning and knowledge transfer of the theoretical components of the training programme into clinical practice.

Cowell, I., O'Sullivan, P., O'Sullivan, K., Poyton, R., McGregor, A., & Murtagh, G. (2019, Mar). The perspectives of physiotherapists on managing nonspecific low back pain following a training programme in cognitive functional therapy: A qualitative study. *Musculoskeletal Care, 17*(1), 79-90. https://doi.org/10.1002/msc.1370
